# Particularities of anaemia in framework of systemic lupus erythematosus

**DOI:** 10.1186/1479-5876-10-S3-P43

**Published:** 2012-11-28

**Authors:** Elena Samohvalov, Lucia Mazur-Nicorici, Ninel Revenco, Minodora Mazur

**Affiliations:** 1 Republic of Moldova Chisinau SMPhU ”Nicolae Testemitanu”, Chisinau, Moldova

## Background

Hematological manifestations are frequently seen in systemic lupus erythematosus (SLE). Anaemia, leucopenia and thrombocytopenia may result from bone marrow failure or excessive peripheral cell destruction, both of which may be immune mediated. Anaemia is a common manifestation in patients with systemic lupus erythematosus. The most common forms of anaemia, in these patients, are autoimmune hemolytic anemia (AHA), iron-deficiency anaemia (IDA), anaemia of chronic disease (ACD), drug-induced myelotoxicity, anaemia of chronic renal failure and rarely types such as pure red cell aplasia, B12 deficiency anemia and myelofibrosis.

## Aim of study

To evaluate the types of anaemia in systemic lupus erythematosus and their association with activity of the disease and their impact in quality of life.

## Methods

We examined 86 SLE patients by ACR, 1997 criteria, from them 59 were with hematological abnormalities. Disease duration was calculated from the time at least four and more ACR criteria were fulfilled. Anti-ds DNA antibodies, erythropoietin and ferritin levels were measured at baseline. Disease activity was assessed with the Systemic Lupus Activity Measure (SLAM).

## Results

The cohort consisted of 59 women with mean age 31.3 ± 1.9 (range 13-68) years and mean disease duration 8 years - 94.56 (range one month to 96 months). Hematological modifications were manifested in 65 (75.6%). Anaemia was more common maniferstation. After the investigations we determined the following forms of anemia: the anemia of chronic disease (ACD) n = 34 (57.6%), iron deficiency anemia (IDA) n = 20 (33.9%), autoimmune hemolytic anemia (AHA) n = 3 (5.1%) and other forms (anaemia of chronic renal failure) causes n = 2 (3.4%). Leucopenia was present at 18 (30.5%) patients, limphopenia at 19 (32.2%) patients and thrombocitopenia at 13 (22.0%) patients. Assessment of disease activity by SLAM showed that low disease activity was determined in 12 (20.3%) patients, moderate activity was found in 36 (61.0%) and high activity - in only 11 (18.6%) patients. This demonstrated that in examined patients was predominant moderate activity 61.0% cases and ACD 57.6%.

## Conclusions

Anaemia is a common manifestation in SLE and it is closely associated with moderate disease activity.

These associations are observed after one year of evolution of systemic lupus erythematosus. Different levels of anaemia may be used to monitor the disease activity.

